# Determinants of hypertension among Bhutanese adults: evidence from a national WHO STEPS survey

**DOI:** 10.1038/s41598-026-35911-w

**Published:** 2026-01-16

**Authors:** Kuenzang Chhezom, Kinley Wangdi

**Affiliations:** 1 Faculty of Undergraduate Medicine, Khesar Gyalpo University of Medical Sciences of Bhutan, Thimphu, Bhutan; 2 HEAL Global Research Centre, Health Research Institute, Faculty of Health, University of Canberra, Bruce, ACT 2617 Australia; 3 National Centre for Epidemiology and Population Health, College of Law, Governance and Policy, Australian National University, Acton, ACT 2601 Australia; 4 Office of the President, Khesar Gyalpo University of Medical Sciences of Bhutan, Thimphu, Bhutan

**Keywords:** Bhutan, Hypertension, Risk factors, Regression, Survey, STEP, NCD, Bayesian, Network, Cardiology, Diseases, Health care, Medical research, Risk factors

## Abstract

Hypertension is an emerging public health problem in Bhutan due to epidemiological and nutritional transitions in the last two decades. This study aimed to quantify risk factors of hypertension in Bhutan using nationally representative data. This was a secondary data analysis of a nationally representative World Health Organization Non-Communicable Disease STEP-wise Survey of Bhutan carried out in 2019. Multivariable logistic regression analysis was conducted to identify the risk factors of hypertension in the population 40–69 years age group. The Bayesian network (BN) analysed the influencing factors on the prevalence of hypertension. Of the 2,574 individuals aged 40–69 years sampled, 56.8% were women. The prevalence of hypertension was 44.3% (1,140). In multivariable logistic regression, participants in 55–59 and 60–64 years were 44% (adjusted odds ratio [AOR] = 1.44; 95% confidence interval [CI] 1.06, 2.14) and 65% (AOR = 1.65; 95% CI 1.17, 2.42) higher odds of developing hypertension than 40–44 years old. Compared to non-formal education, certificate and bachelor, and year 12 educated were 77% (AOR = 1.77; 95% CI 1.01, 3.12) and 54% (AOR = 1.54; 95% CI 1.02, 2.32) odds of reporting hypertension. The higher wealth index had lower odds of hypertension than those in the lower wealth index. Alcohol use was associated with developing hypertension (AOR = 1.73; 95% CI 1.39, 2.15) compared to non-drinkers. Those who chewed betel quid were 38% (AOR = 0.62; 95% CI 0.50, 0.76) less odds of reporting hypertension than those who did not chew betel quid. Individuals with obesity (AOR = 2.39; 95% CI 1.84, 3.12) and overweight (AOR = 1.44; 95% CI 1.04, 1.98) had more than twice and 44% higher odds of developing hypertension compared to those with normal body mass index (BMI). Borderline (AOR = 2.0; 95% CI 1.05, 3.81) and high cholesterol levels (AOR = 1.84; 95% CI 1.32, 2.57) were associated with an increased odds of hypertension compared to normal blood cholesterol levels. The conditional probability of hypertension using BN showed that obese and border blood cholesterol was associated with an increased odds of hypertension. While use alcohol use and chewing betel quid lowered the odds of hypertension. These findings highlight the need for targeted hypertension prevention strategies that address both behavioural and socioeconomic factors such as older age, alcohol use, pre-obesity and obesity, and high cholesterol levels. Therefore, the Ministry of Health, Bhutan should implement a comprehensive prevention and management program that include both clinical care and non-pharmacological strategies such as promoting healthy lifestyles, maintaining optimal body weight, and conducting regular screenings for hypertension and related conditions like high cholesterol.

**Supplementary Information:**

The online version contains supplementary material available at 10.1038/s41598-026-35911-w.

## Introduction

Globally, around 1.28 billion adults aged 30–79 years had been diagnosed with hypertension in 2008 of which two-thirds are living in low and middle-income countries^1,2^. Hypertension (systolic and/or diastolic blood pressure equal to or above 140/90 mmHg)^3^, is responsible for at least 45% of deaths due to cardiovascular disease (CVD) and 51% of deaths are due to stroke^4^. Importantly, hypertension is the leading modifiable risk factor for several CVDs, such as heart failure, ischemic heart diseases, and peripheral arterial diseases^2,5^.

A number of modifiable and non-modifiable risk factors of hypertension have been identified. Understanding these associated risk factors of hypertension is useful for both prevention and management. Modifiable risk factors are those that individuals can alter through lifestyle changes and medical interventions. Controlling or modifying these factors can significantly reduce the risk of developing hypertension. Diet is an important modifiable risk factor, consumption of a high-salt diet^6^, saturated fats and trans fats contribute to hypertension^7^. Other modifiable risk factors are alcohol consumption^8–10^, tobacco use^11^, and physical inactivity^12^. Even though non-modifiable risk factors cannot be changed or modified, understanding them is important for identifying at-risk individuals for initiating intervention strategies. Among non-modifiable risk factors, age and gender are independent predictors of future hypertension and are directly correlated with rising blood pressure^13,14^. The other risk factors of hypertension are ethnicity, diabetes mellitus, obesity, and kidney disease^10,15–18^.

In the last two decades, due to development, Bhutan has been undergoing an epidemiological and nutritional transition^19^. This has led to rising disposable income levels with a shift from traditional high-carbohydrate, low-fat diets towards diets with a lower carbohydrate and a higher proportion of saturated fat, sugar and salt, and less physical activities (due to rapid urbanization)^20^. In 2007, the prevalence rate of hypertension in Bhutan was 17.1%^21^, while it was 26% in the capital city Thimphu during the same period^22^. However, the national prevalence increased to 35.7% in 2014^23^. There was a slight decrease in the prevalence of hypertension to 28% in 15–69 years in 2019^24^ and 60.3% of ≥ 40 years old had very low laboratory-based 10 years CVD risks in Bhutan^25^. The risk factors of hypertension could have shifted over the years, and it is important to identify the risk factors on hypertension using the latest nationally representative data in Bhutan for prevention programs.

Bayesian networks (BNs) offer opportunities to analysis the relationship between the risk factors and outcome variable by capturing the dependence relationship between the selected variables^26^. In the BN method, a directed acyclic graph (DAG) is constructed to intuitively reflect the potential relationship between factors, and a conditional probability distribution table is used to reflect the strength of association^27^. Unlike logistic regression, BNs allow for estimating the subsequent probability of any target variable given any set of conditioning variables, which can predict the probability of having hypertension in a more flexible manner^28,29^. Therefore, the primary aim of this study was to determine the prevalence of hypertension among Bhutanese adults, explore the associated risk factors, and assess the conditional probability of hypertension based on these risk factors using BN.

## Methods

### Study data source and study site

This was a secondary data analysis of nationally representative data based on World Health Organization’s (WHO) approved STEP-wise survey method. A nationwide cross-sectional study was carried out in 20 districts of Bhutan in April 2019^24^. A target population of 15–69 years were represented by a sample size of 5,575. The survey used a multistage cluster sampling to select the participants with a mix of probability proportionate to size (PPS) and systematic random sampling based on the sampling frame from the Population and Housing Census of Bhutan 2017. “*Gewogs*”, or sub-districts (rural setting) and *thromdes*, or towns (urban setting) were the primary sampling unit (PSU) for this survey covering all 20 districts. Eighty-eight PSUs- 55 and 33 from rural and urban settings were selected using Probability Proportionate to Size (PPS). A total of 352 SSUs (220 from rural and 132 from urban) from 4 Secondary Sampling Units (SSU) were selected from each PSU. For every census block, 16 households were selected using a circular systematic random sampling method. Using the Kish sampling method, only one eligible individual per household was selected randomly^24^. The questionnaire consisted of three steps for measuring the non-communicable disease (NCD) risk factors. In step 1, interviewer administered questionnaire to the one eligible individual from each household. In step 2, physical measurements were taken, and in step 3, biochemical measurements were taken from the participants. A WHO developed structured questionnaire was used to interview study participants followed by physical and biochemical measurements, respectively^24^. Prior to the data collection, five days training was provided to the enumerators and the supervisors. It was followed by pretesting of the questionnaire for two days covering at least 32 interviews in the non-sampled areas^24^.

For this study, individual-level data of ≥ 40 years were extracted from 5,575 surveyed records, and the outcome of interest was hypertensive because of higher risk of hypertension in the older population. A record of 3,001 individual data was deleted from the final data set (2,986 were < 40 years and 15 missing age) and records of 2,574 individuals were retained for analysis. Independent variables were sex, age, education level, residence type defined as urban or rural, wealth index, level of exercise, fruit and vegetable servings in a week, current alcohol user, current betel quid chewing, body weight, diabetes, and blood cholesterol level.

An average of two consecutive blood pressure (BP) readings of both systolic and diastolic BP was used to calculate the blood pressure. According to the WHO, hypertension was defined as individuals with an average measured systolic BP ≥ 140 mmHg or diastolic BP ≥ 90 mmHg, or who reported having been diagnosed with hypertension or receiving BP-lowering treatment^3,30^. Smokers were defined as those participants who smoked ≥ 1 cigarette/day in last 6 months. Alcohol use was defined as drinking alcohol at least 1 time/week, with an alcohol intake of 50 g (used showcard) or more for six consecutive months^24^. A total of 924 participants never used alcohol in their life so were assigned as currently not using alcohol. Exercise was defined as vigorous-intensity activity that causes large increases in breathing or heart rate such as carrying or lifting heavy loads, digging or construction work, cutting wood, and mask dance (*cham*) for at least 10 min continuously. Body mass index (BMI) was calculated as body weight in kg divided by height in meters square. BMI was classified under (< 18.5 kg/m^2^), normal (18.5–22.9 kg/m^2^), overweight (23.0–24.9 kg/m^2^), and obesity (≥ 25.0 kg/m^2^) for the Asia Pacific population^31^. Blood cholesterol was stratified as normal (min-5.19 mmol/L), borderline (5.2–6.19 mmol/L), and high (≥ 6.2 mmol/L). Diabetes was defined as a fasting plasma glucose value ≥ 7.0 mmol/L [126 mg/dl] or being on medication for raised blood glucose^32^.

### Logistic regression

Sampling weights used to adjust for the sample complex design. From the univariable analysis, covariates with *p* value of 0.2 or less were fitted into the final model. In the logistic multivariable regression models, sample weights of respective survey data sets (individual modules) were used to get nationally representative estimates. Adjusted odds ratios (AOR), their 95% confidence intervals (CI), and *p* values were calculated as appropriate and < 0.05 was considered significant (2-sided). The receiver operating characteristic (ROC) curve was plotted to assess the accuracy and reliability of covariates in multivariable logistic regression in predicting the prevalence of hypertension. All explanatory variables in the multivariable model were tested for multicollinearity using a variance inflation factor (VIF), where VIF < 10 was considered a good fit for regression analysis (Supplementary Table 1). The analysis was performed in R 4.4.2 (R Core Team, 2023) using RStudio (Posit team, 2025).

### Bayesian belief network (BBN)

A Bayesian network (BN) is a DAG that represents a set of variables and their conditional dependencies through defined probabilities^27^. BN models are used to represent knowledge and reasoning under uncertainty. The DAG is represented by a graphical structure with “parent” and “child” nodes linked by arrows showing the presence of probabilistic conditional dependence between these two variables^28,33^. The conditional probability distributions (i.e., priori or unconditional, conditional, and posterior probabilities) describe the relationships between these nodes^28,33^. A BN model is based on Bayes’ theorem of probability theory to propagate information between nodes. Bayes’ theorem illustrates how prior knowledge about a given hypothesis X is updated by observed evidence Y as shown below:

$$\:P\left(X|Y\right)=\frac{P\left(X\right)*P\left(Y\right|X)}{P\left(Y\right)}$$

where *P(X)*, is the prior probability of the hypothesis *X* (i.e., the likelihood that *X* will be in a particular state, prior to consideration of any evidence), *P(Y|X)* is the conditional probability (i.e., the likelihood of the evidence, given the hypothesis to be tested); and *P(X|Y)* is the posterior probability of the hypothesis (i.e., the likelihood that *X* is in a particular state, conditional on the evidence provided). This equation showing probabilities gives an explicit representation of uncertainties^34^.

### BN for hypertension risk factors

In this study, the outcome of interest was hypertension among adult Bhutanese ≥ 40 years. The correlates of hypertension were based on the logistic regression. Seven variables including age, education, wealth index, current alcohol, betel quid chewing, BMI, and blood cholesterol were included for BN analysis. Using the outcome variable, data were randomly partitioned into training and test datasets. The training consisted of 80% (2,017) of the total records (2,574) and the rest (557) were assigned to the testing set. The learning of the conditional probability tables (CPTs) was conducted using an expectation–tabu search algorithm, optimized with the Bayesian Dirichlet equivalent uniform (BDeu) scoring metric. The BDeu scoring metric is a method used to evaluate Bayesian network structures based on observed data^35^. It assumes a uniform prior over both network structures and their parameters, meaning no structure or parameter values are favoured before seeing the data. The score is calculated using the marginal likelihood of the data, incorporating counts of variable configurations and a hyperparameter called the equivalent sample size (ESS), which determines the influence of the prior^35,36^. BDeu is commonly applied in greedy search algorithms like hill-climbing for structure learning, and it ensures score equivalence- assigning the same score to network structures that encode the same conditional independence relationships^37^. This process was implemented in R using the ‘bnlearn’ package and was applied to a training dataset comprising 2,017 observations. The BN was compiled using Netica software version 7.01 (Norsys Software Corp., Vancouver, Canada).

## Results

### Descriptive results

Of the 2,574 individuals aged 40–69 years, 56.8% (1,462) were females. Of the total individuals, 72.4% (1,861) and 13.5% (347) had no or non-formal education and primary education, respectively. Around 72.7% (1,872) lived in rural areas and a third of the study population (27.9%, 717) were in the least wealthy quartile. More than half of the participants (1,321) exercised regularly. A quarter (24.7%, 636) and 85.0% (2,187) of the surveyed participants reported consuming 4–7 servings of fruits and vegetables per week, respectively. Current smoker among the study participants were 3.3% (86). Current alcohol use and betel quid chewing were reported in 41.5% (1,068), and 54.5% (1,402) of participants. More than 17% (451) and 56.3% (1,448) were overweight and obese. The proportion of respondents with diabetes and high cholesterol levels was 3.6% (91) and 2.6% (66) (Table 1).

**Table 1:** Sociodemographic characteristics of respondents aged 40–69 years in Bhutan from WHO NCD STEPS Survey, 2019.

Characteristics		Total participants	Hypertensive
		Number (2,574) (%)	Number (1,140) (%)
Sex	Men	1,112 (43.2)	533 (46.8)
	Women	1,462 (56.8)	607 (53.2)
Age	40–44	626 (24.3)	238 (20.9)
	45–49	544 (21.1)	221 (19.4)
	50–54	467 (18.1)	207 (18.2)
	55–59	356 (13.8)	181 (15.9)
	60–64	320 (12.4)	158 (13.9)
	65–69	261 (10.1)	135 (11.8)
Education^&^	NFE	1,861 (72.4)	803 (70.5)
	CB^ϕ^	120 (4.7)	69 (6.1)
	Up to year 12	200 (7.8)	103 (9.0)
	Up to Primary	347 (13.5)	143 (12.6)
	Others	44 (1.7)	21 (1.8)
Urban	No	1,872 (72.7)	809 (71.0)
	Yes	702 (27.3)	331 (29.0)
Wealth index	Least wealth	717 (27.9)	344 (30.2)
	Lower	556 (21.6)	227 (19.9)
	Middle	514 (20.0)	207 (18.2)
	Upper	403 (15.7)	185 (16.2)
	Wealthiest	384 (14.9)	177 (15.5)
Exercise	No	1,253 (48.7)	600 (52.6)
	Yes	1,321 (51.3)	540 (47.4)
Fruit serving	No serving	686 (26.7)	292 (25.6)
	1–3 serving	1,252 (48.6)	561 (49.2)
	4–7 servings	636 (24.7)	287 (25.2)
Vegetable serving per week	No serving	63 (2.4)	24 (2.1)
	1–3 serving	324 (12.6)	151 (13.2)
	4–7 servings	2,187 (85.0)	965 (84.6)
Alcohol use	No	1,506 (58.5)	596 (52.3)
	Yes	1,068 (41.5)	544 (47.7)
Current smoker	No	2,488 (96.7)	1,101 (96.6)
	Yes	86 (3.3)	39 (3.4)
Betel chewing	No	1,172 (45.5)	580 (50.9)
	Yes	1,402 (54.5)	560 (49.1)
BMI*	Under	62 (2.4)	24 (2.2)
	Normal	613 (23.8)	213 (18.7)
	Overweight	451 (17.5)	184 (16.1)
	Obese	1,448 (56.3)	718 (63.0)
Diabetes^**^	No	2,432 (96.4)	1,090 (94.5)
	Yes	91 (3.6)	50 (4.5)
Cholesterol^††**^	Normal	2,210 (87.6)	942 (84.1)
	Borderline	247 (9.8)	135 (12.1)
	High	66 (2.6)	43 (3.8)

^†^NCD- non communicable disease; WHO: World Health Organization

*BMI- body mass index; Under (< 18.5 kg/m^2^); Normal (18.5–22.9 kg/m^2^); Overweight (23.0–24.9 kg/m^2^); Obese (> 24.9 kg/m^2^).

^††^borderline blood cholesterol (5.2–6.19 mmol/L); ^††^high blood cholesterol (≥ 6.2 mmol/L).

^ϕ^CB- Certificate and bachelor; ^¶^NFE- no or non-formal education.

^&^no education in 2 and 1 individuals in total and hypertensive patients.

^**^51 cases have no blood sugar and cholesterol.

The national prevalence of hypertension in Bhutan was 44.3% (1,140/2,574). It was found that a higher proportion of women (53.2%, 607) had hypertension compared to males. Among the hypertensive, 70.5% (803) of the individuals had no or NFE education, 71.0% (809) of the individuals were living in rural areas and 30.2% (334) belonged to the category of least wealth index. Nearly half (561) and 13.2% (151) of hypertensive patients had one to three servings of fruits and vegetables in a typical week. Forty-eight (544), 3.4% (39), and 49.1% (560) used alcohol, smoked, and chewed betel quid, respectively. Around 63.0% (718) were obese and 84.1% (942) had normal cholesterol levels (Table 1).

### Logistic regression results

**Fig. 1:**
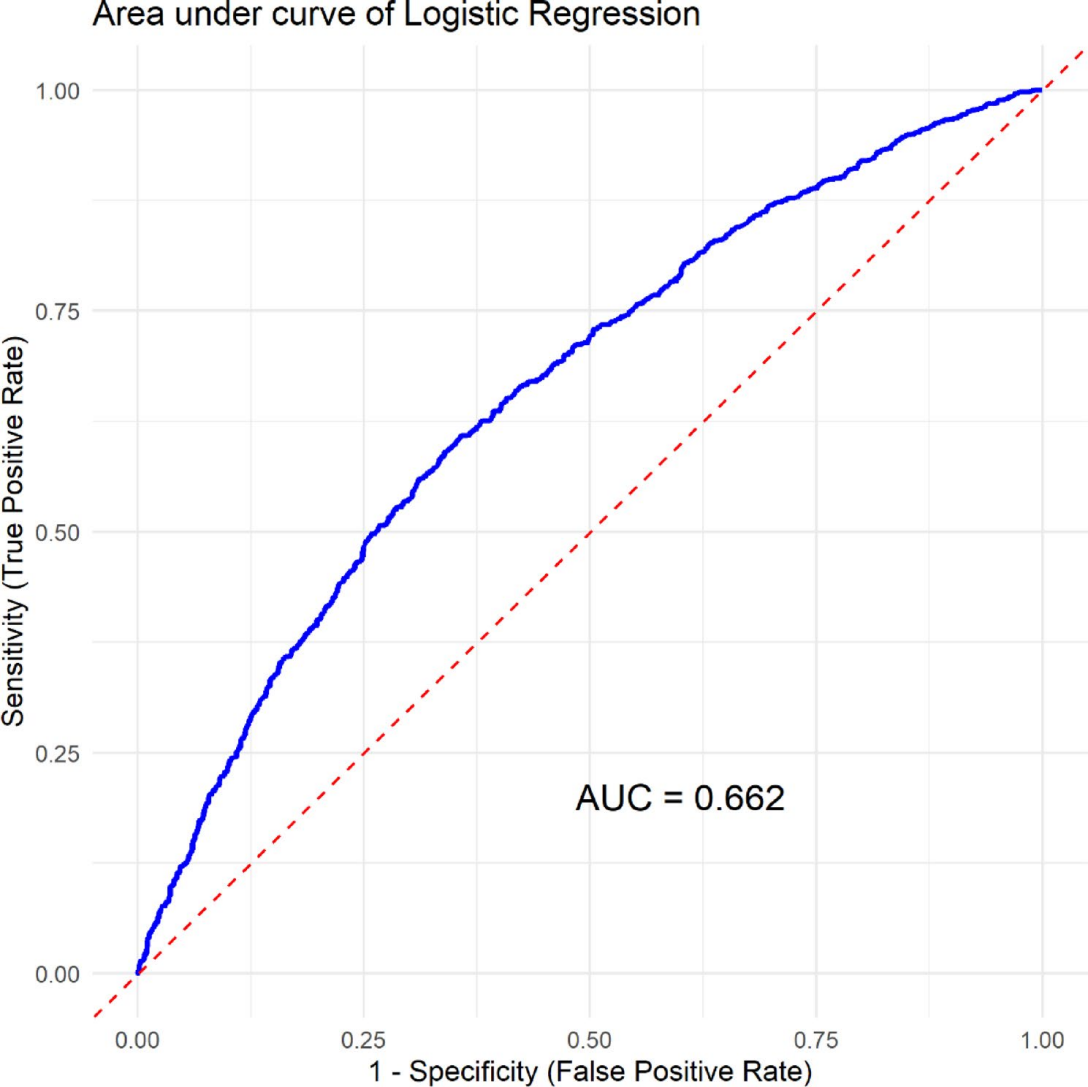
Receiver operating characteristic (ROC) curve analysis of sex, age, education, wealth index, urban residents, exercise, vegetable consumption, alcohol use, betel quid chewing, diabetes, BMI and blood cholesterol for predicting hypertension.

In univariable analysis, sex, age, education, wealth index, urban residents, exercise, vegetable consumption, alcohol use, betel quid chewing, diabetes, BMI and cholesterol were significant at *p*-value of 0.2 (Table 2). The above variables were fitted into multivariable logistic regression. After controlling other co-variates, participants in 55–59 and 60–64 years were 44% (AOR = 1.44; 95% CI 1.06, 2.14) and 65% (AOR = 1.65; 95% CI 1.17, 2.42) higher odds of developing hypertension compared to those aged 40–44 years. Compared to non-formal education, certificate and bachelor, and year 12 educated were 77% (AOR = 1.77; 95% CI 1.01, 3.12) and 54% (AOR = 1.54; 95% CI 1.02, 2.32) odds of reporting hypertension, respectively. Members of households in the wealthiest (AOR = 0.57, 95% CI 0.36, 0.90), middle (AOR = 0.59; 95% CI 0.43, 0.81), and lower (AOR = 0.72; 95% CI 0.54, 0.96) wealth index had lower odds of hypertension than those individuals in the lowest wealth index. Alcohol use was associated with developing hypertension (AOR = 1.73; 95% CI 1.39, 2.15) compared to non-drinkers. Those who chewed betel quid were 38% (AOR = 0.62; 95% CI 0.50, 0.76) less odds of reporting hypertension than those who did not chew betel quid. Individuals with obesity (AOR = 2.39; 95% CI 1.84, 3.12) and overweight (AOR = 1.44; 95% CI 1.04, 1.98) were twice and 87% more likely to develop hypertension compared to those with normal body mass index. Borderline (AOR = 2.0; 95% CI 1.05, 3.81) and high cholesterol levels (AOR = 1.84; 95% CI 1.32, 2.57) were associated with an increased odds of hypertension compared to normal blood cholesterol levels (Table 2). The predictive performance (AUC) of the model was 0.662 suggesting model can fair discrimination ability to correctly distinguish between positive and negative cases about 66.2% of the time (Fig. 1).

**Table 2:** Factors associated with hypertension among respondents aged 40–69 years in Bhutan from WHO NCD STEPS Survey, 2019.

Characteristic		OR (95% CI)	*p*-value	AOR (95% CI)	*p*-value
Sex	Men	Ref		Ref	
	Women	1.23 (1.01, 1.49)	0.037	1.18 (0.94, 1.47)	0.2
Age groups (years)	40–44	Ref		Ref	
	45–49	0.94 (0.71, 1.24)	0.6	0.96 (0.72, 1.29)	0.8
	50–54	1.2 (0.89, 1.61)	0.2	1.34 (0.98, 1.81)	0.062
	55–59	1.29 (0.92, 1.80)	0.14	1.44 (1.06, 2.14)	0.043
	60–64	1.34 (0.96, 1.89)	0.09	1.65 (1.17, 2.42)	0.008
	65–69	1.4 (0.98, 2.00)	0.065	1.45 (0.99, 2.13)	0.053
Education	NFE	Ref		Ref	
	CB	2.03 (1.29, 3.20)	0.002	1.77 (1.01, 3.12)	0.048
	Others	1.12 (0.57, 2.21)	0.7	1.01 (0.51, 2.03)	> 0.9
	Up to 12 years	1.47 (1.03, 2.09)	0.034	1.54 (1.02, 2.32)	0.042
	Up to Primary	1.09 (0.81, 1.45)	0.6	1.24 (0.91, 1.69)	0.2
Wealth index	Least wealth (Q1)	Ref		Ref	
	Lower (Q2)	0.7 (0.53, 0.92)	0.01	0.72 (0.54, 0.96)	0.026
	Middle (Q3)	0.62 (0.47, 0.83)	0.001	0.59 (0.43, 0.81)	0.001
	Upper (Q4)	1.03 (0.77, 1.38)	0.9	0.72 (0.49, 1.05)	0.089
	Wealthiest (Q5)	1.04 (0.78, 1.41)	0.8	0.57 (0.36, 0.90)	0.016
Urban	Rural	Ref		Ref	
	Urban	1.27 (1.02, 1.57)	0.031	1.13 (0.84, 1.52)	0.4
Exercise	No	Ref		Ref	
	Yes	0.74 (0.61, 0.90)	0.002	0.84 (0.66, 1.05)	0.13
Fruit serving per week	No	Ref			
	1–3 servings	1.11 (0.88, 1.40)	0.4		
	4–7 servings	1.04 (0.79, 1.36)	0.8		
Vegetable serving per week	No	Ref		Ref	
	1–3 servings	1.42 (0.82, 2.49)	0.2	1.5 (0.68, 3.30)	0.3
	4–7 servings	1.28 (0.77, 2.18)	0.3	1.48 (0.70, 3.12)	0.3
Alcohol use	No	Ref		Ref	
	Yes	1.6 (1.31, 1.94)	< 0.001	1.73 (1.39, 2.15)	< 0.001
Current smoker	No	Ref			
	Yes	1 (0.60, 1.65)	> 0.9		
Betel chewing	No	Ref		Ref	
	Yes	1.64 (0.72, 3.85)	0.2	0.62 (0.50, 0.76)	< 0.001
Diabetes	No	Ref		Ref	
	Yes	1.66 (1.00, 2.74)	0.05	1.34 (0.79, 2.29)	0.3
BMI^**^	Normal	Ref		Ref	
	Obese	2.05 (1.61, 2.74)	< 0.001	2.39 (1.84, 3.12)	< 0.001
	Overweight	1.31 (0.97, 1.77)	0.085	1.44 (1.04, 1.98)	0.027
	Under weight	1.28 (0.70, 2.35)	0.4	1.25 (0.67, 2.33)	0.5
Cholesterol^††^	Normal	Ref		Ref	
	High	1.86 (1.33, 2.59)	< 0.001	1.84 (1.32, 2.57)	< 0.001
	Borderline	2.21 (1.23, 3.97)	0.008	2.0 (1.05, 3.81)	0.034

NCD: non communicable disease. WHO: World Health Organization

OR- odds ratio: AOR- adjusted odds ratio; CI- confidence interval; Ref- reference; NFE- non-formal education; ^**^BMI- body mass index; Under (< 18.5 kg/m^2^); Normal (18.5–22.9 kg/m^2^); Overweight (23.0–24.9 kg/m^2^); Obese (> 24.9 kg/m^2^); ^††^borderline blood cholesterol (5.2–6.19 mmol/L); ^††^high blood cholesterol (≥ 6.2 mmol/L).

CB- Certificate and bachelor.

### Bayesian network model for hypertension

**Fig. 2:**
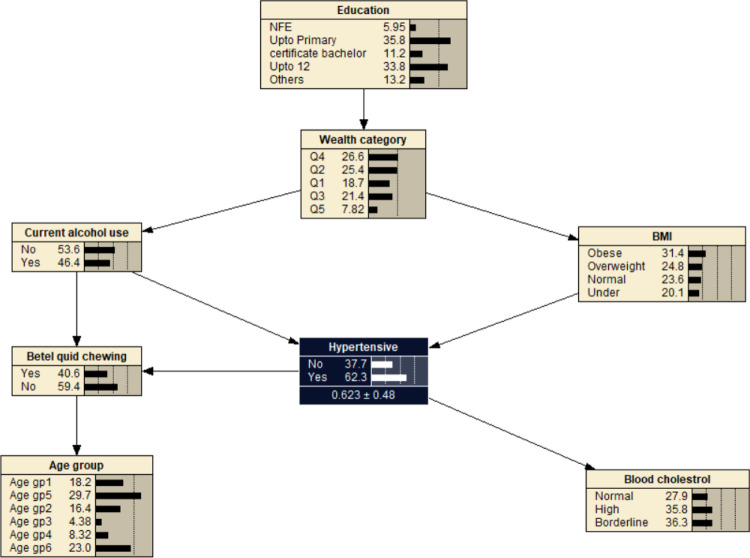
The Bayesian network model showing hypertension and related factors. BMI- body mass index: Under (< 18.5 kg/m^2^); Normal (18.5–22.9 kg/m^2^); Overweight (23.0–24.9 kg/m^2^); Obese (> 24.9 kg/m^2^). Age gp1: 40–44 years; Age gp2: 45–49 years; Age gp3: 50–54 years; Age gp4: 55–59 years; Age gp5: 60–64 years; Age gp6: 65–69 years. Wealth index: Q1- Least wealth; Q2- Lower; Q3- Middle; Q4- Upper; Q5- Wealthiest. Cholesterol: borderline cholesterol (5.2–6.19 mmol/L); high (≥ 6.2 mmol/L). Education: NFE- no or non-formal education.

The significant variables from the multivariable logistic regression were used to construct a simple BN model. The significant variables were age, education, wealth index, alcohol use, betel quid chewing, BMI, and blood cholesterol. It consisted of 8 nodes and 9 arcs with education and wealth index as parent nodes of main factors associated with hypertension. Each node represented one variable and the probabilistic dependencies were connected by arc between nodes. The resulting probabilistic model can be used to quantitatively analyse the impact of these factors on hypertension by computing the conditional probabilities *P*(*y*|*xi*). The prior probability of hypertension was *P*_*(hypertension)*_ = 0.623 (Fig. 2).

**Fig. 3:**
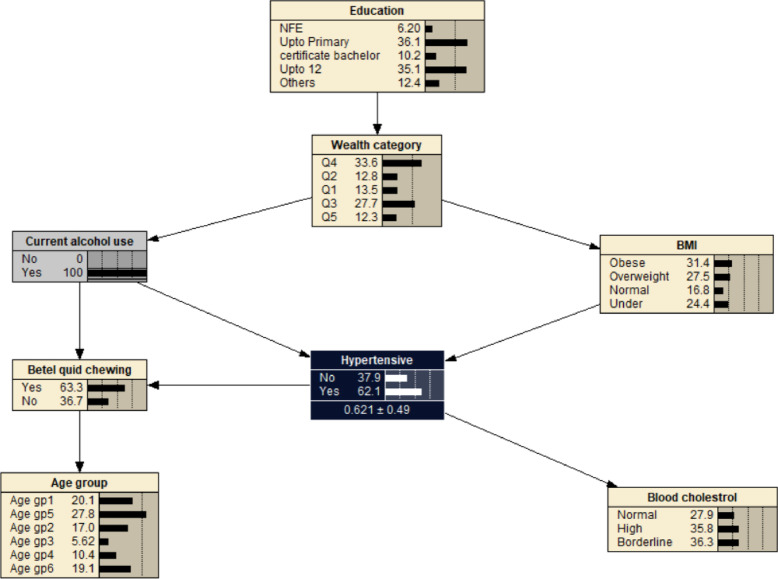
The Bayesian network for hypertension for all participants reported using alcohol. BMI- body mass index: Under (< 18.5 kg/m^2^); Normal (18.5–22.9 kg/m^2^); Overweight (23.0–24.9 kg/m^2^); Obese (> 24.9 kg/m^2^). Age gp1: 40–44 years; Age gp2: 45–49 years; Age gp3: 50–54 years; Age gp4: 55–59 years; Age gp5: 60–64 years; Age gp6: 65–69 years. Wealth index: Q1- Least wealth; Q2- Lower; Q3- Middle; Q4- Upper; Q5- Wealthiest. Cholesterol: borderline cholesterol (5.2–6.19 mmol/L); high (≥ 6.2 mmol/L). Education: NFE- no or non-formal education.

**Fig. 4:**
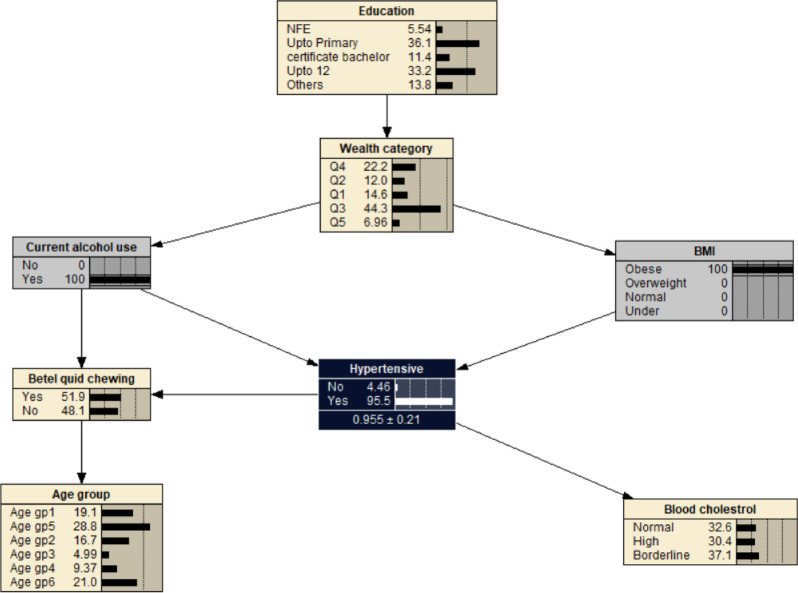
The Bayesian networks network for hypertension for all the participants reported using alcohol and being obese. BMI- body mass index: Under (< 18.5 kg/m^2^); Normal (18.5–22.9 kg/m^2^); Overweight (23.0–24.9 kg/m^2^); Obese (> 24.9 kg/m^2^). Age gp1: 40–44 years; Age gp2: 45–49 years; Age gp3: 50–54 years; Age gp4: 55–59 years; Age gp5: 60–64 years; Age gp6: 65–69 years. Wealth index: Q1- Least wealth; Q2- Lower; Q3- Middle; Q4- Upper; Q5- Wealthiest. Cholesterol: borderline cholesterol (5.2–6.19 mmol/L); high (≥ 6.2 mmol/L). Education: NFE- no or non-formal education.

**Fig. 5:**
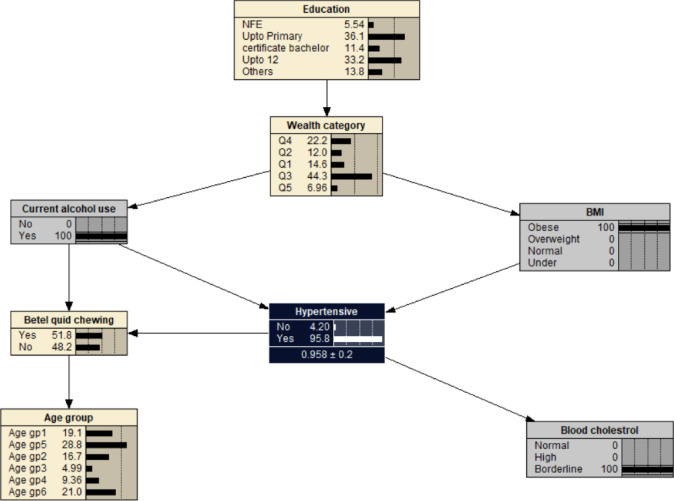
The Bayesian network for hypertension for all the participants reported using alcohol, being obese and borderline blood cholesterol. BMI- body mass index: Under (< 18.5 kg/m^2^); Normal (18.5–22.9 kg/m^2^); Overweight (23.0–24.9 kg/m^2^); Obese (> 24.9 kg/m^2^). Age gp1: 40–44 years; Age gp2: 45–49 years; Age gp3: 50–54 years; Age gp4: 55–59 years; Age gp5: 60–64 years; Age gp6: 65–69 years. Wealth index: Q1- Least wealth; Q2- Lower; Q3- Middle; Q4- Upper; Q5- Wealthiest. Cholesterol: borderline cholesterol (5.2–6.19 mmol/L); high (≥ 6.2 mmol/L). Education: NFE- no or non-formal education.

**Fig. 6:**
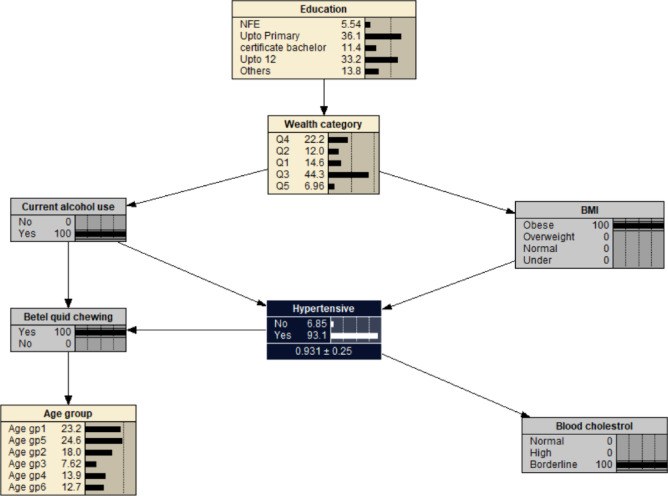
The Bayesian network for hypertension for all the participants reported using alcohol, being obee, borderline blood cholesterol, and chewing betel quid. BMI- body mass index: Under (< 18.5 kg/m^2^); Normal (18.5–22.9 kg/m^2^); Overweight (23.0–24.9 kg/m^2^); Obese (> 24.9 kg/m^2^). Age gp1: 40–44 years; Age gp2: 45–49 years; Age gp3: 50–54 years; Age gp4: 55–59 years; Age gp5: 60–64 years; Age gp6: 65–69 years. Wealth index: Q1- Least wealth; Q2- Lower; Q3- Middle; Q4- Upper; Q5- Wealthiest. Cholesterol: borderline cholesterol (5.2–6.19 mmol/L); high (≥ 6.2 mmol/L). Education: NFE- no or non-formal education.

Alcohol use was associated with decreased probability of developing hypertension at 0.621, i.e. *P*_(*hypertension | alcohol use)*_ = 0.621 (Fig. 3). If the individual was also obese (BMI > 29.9), the likelihood of developing hypertension increased to *P*_(*hypertension | alcohol use, obese)*_ = 0.955 (Fig. 4). The probability of hypertension increased to 0.958 in individuals with borderline cholesterol, *P*_*(hypertension | alcohol use, obese, borderline cholesterol)*_ = 0.958 (Fig. 5). While the probability of hypertension decreased to 0.931 in individuals chewing betel quid, *P*_*(hypertension | alcohol use, obese, border line cholesterol, betel quid use)*_ = 0.931 (Fig. 6). The the area under ROC curve was 0.5389 (Supplementary Fig. 1).

## Discussion

This study has demonstrated that approximately half of adults aged 40–69 years were hypertensive. Non-modifiable risk factor such as age was associated with a higher odds of hypertension. Conversely, people with a higher wealth index and those chewing betel quid were at a lower risk of hypertension. Other important lifestyle risk factors of hypertension were alcohol use, education, overweight, obesity, and abnormal blood cholesterol. The effects of different correlates on the risk of hypertension were tested using BN model.

The prevalence of hypertension in this study was higher than those previously reported^22,38^ but lower than another report from STEP 2014^23^. Hypertension has been identified as an important NCD in Bhutan^39^. Therefore, Bhutan government has rolled out the Service with Care and Compassion Initiative (SCCI) for NCDs as an adaptation of the WHO Package for Essential NCD (PEN) protocol to primary health facilities^40^. Such initiatives are expected to decrease the prevalence of hypertension in the country^41^. However, regular evaluation of such program is needed to identify the barriers such as the training needs of health care providers for successful implementation of the program^41,42^.

Hypertension was positively associated with non-modifiable risk factors including age. Other studies have reported similar findings in the past^43^. This underlying reason for such finding is linked to physiological changes associated with aging, where age-related stiffening of the aorta results in a decreased capacity of the elastic reservoir^14,44^. Therefore, prevention focusing on healthy aging by promoting healthy diets and weight, increased physical activity, and treating hypertension should be prioritized in Bhutan^3^. These interventions become more relevant as the population ages and many pre-hypertensive respondents progress to full hypertension. Some of such initiatives in Bhutan include establishment of open-air gymnasiums.

Modifiable risk factor such as current alcohol use was found to be a risk factor of hypertension. Bhutan ranks as one of the highest alcohol use in the region^45^, which is evident from a large number of alcohol outlets (wholesale, retail, and functioning bars) in the country totalling 5,407^46^. In the year 2015 and 2016, alcohol-related ailments were among the top 10 leading causes of morbidity and mortality in Bhutan^47,48^ resulting in a significant health costs to the government in alcohol-related treatment and management^49^. Therefore, Bhutan should take a holistic public health prevention and control program to address alcohol use and hypertension.

Metabolic co-morbidities including overweight and obesity, and abnormal cholesterol were associated with an increased risk of hypertension. Hypertension and diabetes are closely linked co-morbidities and obesity being a common risk factor for hypertension and diabetes^50–52^. In the last two decades, Bhutan has seen an epidemiological and nutritional transition^19^. This has led to higher disposable income and changing from traditional high-carbohydrate, low-fat diets to diets lower in carbohydrates and higher in saturated fat, sugar, and salt, and lower levels of physical activity^20^. It is estimated that around 40% of those aged 15 and above are overweight or obese^53^. Therefore, these cohort are at greater risk of developing hypertension in future.

People in the higher socio-economic status were at a lower odd of hypertension, like other studies^54,55^. This may be attributed to the fact that individuals with higher incomes typically have more frequent access to quality healthcare services. Additionally, they are often more inclined to engage in health-promoting behaviours, such as regular physical activity, maintaining a balanced diet, and adopting a generally healthier lifestyle. Consequently, those with higher annual household incomes are more likely to reside or work in environments conducive to good health, which may contribute to a delayed onset of hypertension or better management of blood pressure.

The BNs can be employed for predicting disease risk, referred to as BN reasoning. This involves inferring the probability of an unknown variable based on the state of a known variable- hypertension risk. The reasoning process is sequential, evaluating disease risk intensity by analysing changes in the conditional probability of specific factors, highlighting its usefulness in prevention efforts^56^. The inference remains valid even when some information is missing. Moreover, the risk inference process demonstrates that changes in the level of a factor within the network lead to corresponding shifts in conditional probabilities. This reflects the interconnectedness of variables and offers a more nuanced and comprehensive understanding of the relationships between each factor and the disease. Therefore, BNs serve as a valuable complement to traditional logistic regression by better capturing the complexity of disease-related associations^56^.

The BN model indicated that certain demographic and health-related factors are significantly associated with a higher prevalence of hypertension. Individuals who presented with borderline blood cholestrol and high BMI (> 29.9) reported higher proportion of hypertension. While education and those who belong to a higher socio-economic status were parent nodes of main factors associated with hypertension. This evidence suggests a critical need for targeted prevention programs in Bhutan that focus on these identified risk factors. Preventive measures to maintain a healthy body weight (obesity), particularly among those with a lower socio-economic status can potentially lead to greater reductions in hypertension rates. Implementing educational campaigns, promoting healthier lifestyle choices, and providing access to regular health screenings can help mitigate these risks. Overall, a strategic approach that prioritizes these key demographics and health indicators will likely yield significant benefits in managing and preventing hypertension within the population^29^.

This study is subjected to a number of limitations. First, the cross-sectional design limits causality assessment and demands further longitudinal studies. Second, the responses on lifestyle were self-reported. Therefore, they are subjected to probable recall bias. Third, social desirability could have undermined some of the self-reported behavioural risk factors including fruit and vegetable consumption, tobacco use, alcohol use, and physical activity. Despite these limitations, the main strength is the nationally representative data and its associated risk factors.

## Conclusion

The prevalence of hypertension was 44.3% in Bhutanese adults aged 40–69 years. Risk of hypertension were age, education, alcohol usage, metabolic co-morbidities including overweight and obese, and borderline and high blood cholesterol levels. While individuals in the higher wealth quintiles had lower odds of developing hypertension. Borderline blood cholesterol and obese were associated with increased proportion in hypertension. 

Ministry of Health, Bhutan should undertake a hypertension prevention and management program by addressing the identified risk factors. To reduce the burden of hypertension, both non-pharmacological and clinical management should be useful. Non-pharmacological approaches including education on a healthy lifestyle by maintaining a healthy body weight should encouraged. Regular screening programs to detect hypertension and associated co-morbidities such as blood cholesterol should be undertaken.

## Supplementary Information

Below is the link to the electronic supplementary material.

Supplementary Material 1

Supplementary Material 2

## Data Availability

The datasets generated during and/or analysed during the current study are available from the corresponding author on reasonable request.
