# Supplementary figures and images for: Determinants of hypertension among Bhutanese adults: evidence from a national WHO STEPS survey
Source: Sci Rep. 2026 Jan 16;16:5329. doi: 10.1038/s41598-026-35911-w (PMC12881454; doi:10.1038/s41598-026-35911-w)

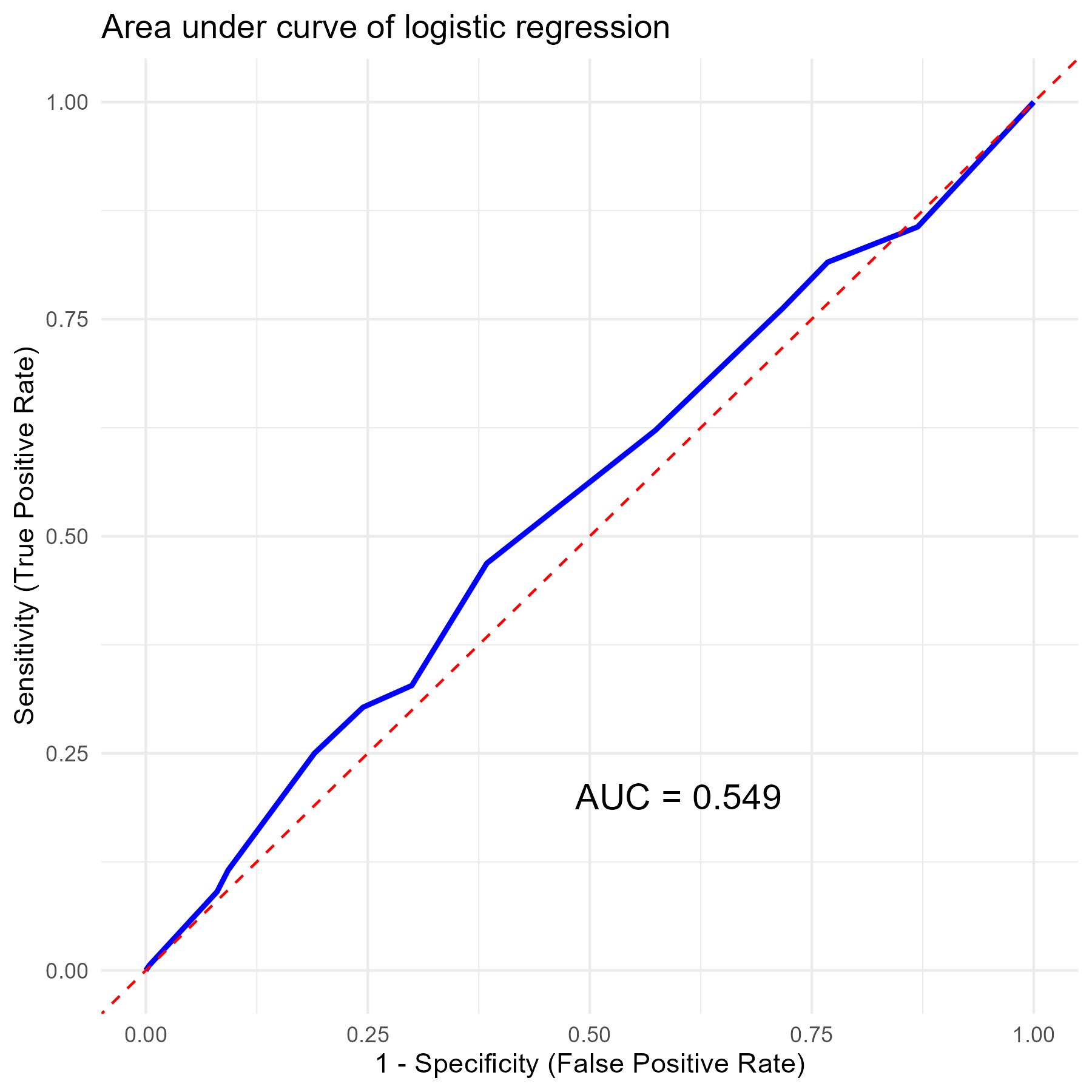

Supplement: Supplementary file 1 — Supplementary Material 1 [file 41598_2026_35911_MOESM1_ESM.tiff]
