# Supplementary material for: Determinants of hypertension among Bhutanese adults: evidence from a national WHO STEPS survey
Source: Sci Rep. 2026 Jan 16;16:5329. doi: 10.1038/s41598-026-35911-w (PMC12881454; doi:10.1038/s41598-026-35911-w)
Supplement: Supplementary file 2 — Supplementary Material 2 [file 41598_2026_35911_MOESM2_ESM.docx]

**Supplementary Materials**

**Supplementary Table 1. Collinearity of factors included in the logistic regression model using variance inflation factor.**

| **Variables** | **VIF** | **1/VIF** |
| --- | --- | --- |
| Sex | 1.250768 | 0.799508782 |
| Age_gp | 1.205836 | 0.82930017 |
| Edu | 1.858328 | 0.538118136 |
| Windex | 2.721246 | 0.367478721 |
| Urban | 1.774643 | 0.563493615 |
| Exercise | 1.399104 | 0.71474315 |
| Veg_serving_week | 1.060361 | 0.943075047 |
| Current_alcohol_use | 1.06703 | 0.937180773 |
| Betel_chewing | 1.024109 | 0.976458561 |
| Diabetes | 1.020215 | 0.980185549 |
| BMI | 1.261752 | 0.792548773 |
| Chole_range | 1.050718 | 0.95173015 |


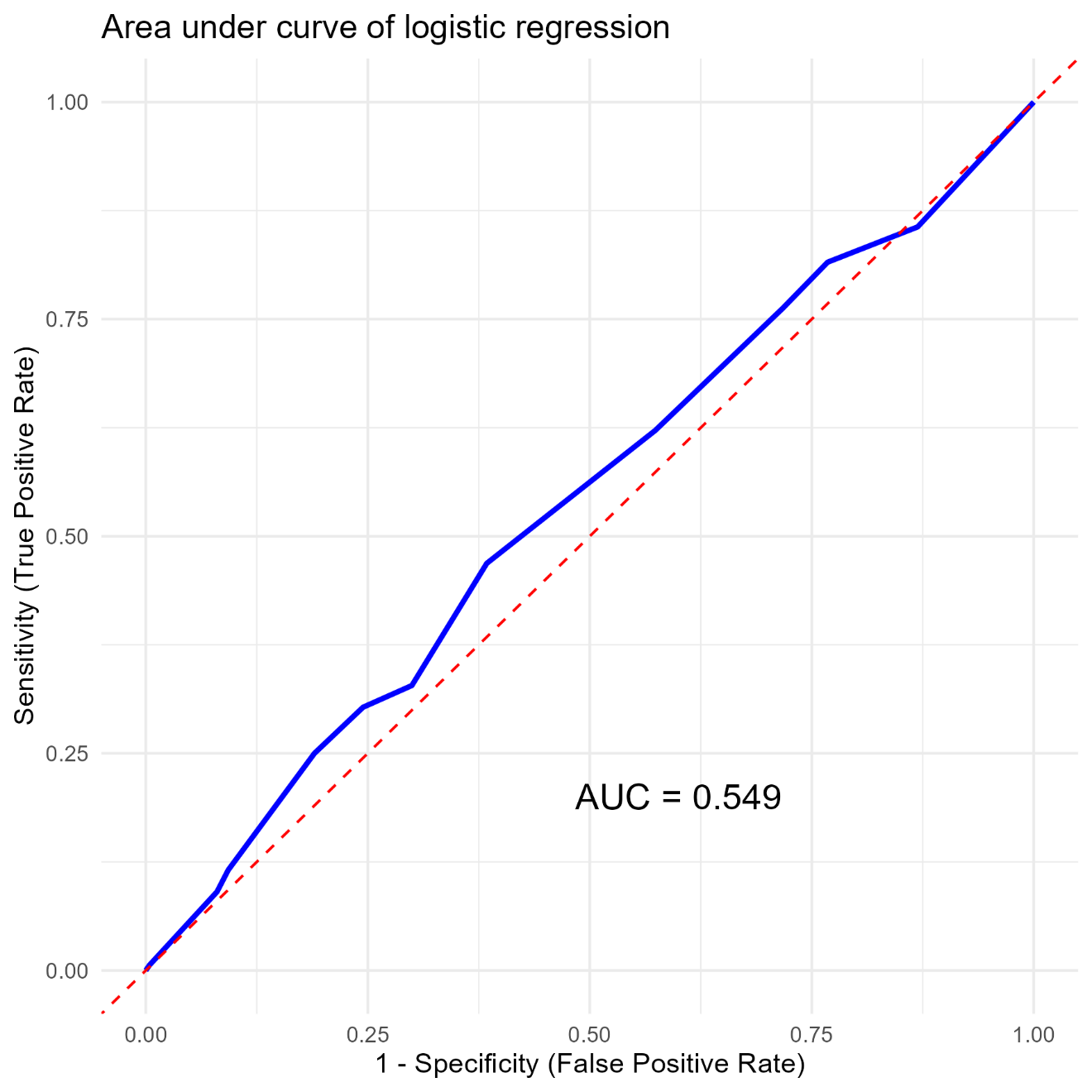


**Supplementary Figure 1 Bayesian networks for hypertension for hypertension for all the participants reported high cholesterol, obesity, alcohol use and least wealth (Q1) stratified by different age groups.**
